# Difference of stage at cancer diagnosis by socioeconomic status for four target cancers of the National Cancer Screening Program in Korea: Results from the Gwangju and Jeonnam cancer registries

**DOI:** 10.1016/j.je.2016.07.004

**Published:** 2017-03-06

**Authors:** Sun-Seog Kweon, Min-Gyeong Kim, Mi-Ran Kang, Min-Ho Shin, Jin-Su Choi

**Affiliations:** a Gwangju/Jeonnam Cancer Registry and Jeonnam Regional Cancer Center, Chonnam National University Hwasun Hospital, Hwasun, South Korea; b Department of Preventive Medicine, Chonnam National University Medical School, Gwangju, South Korea

**Keywords:** National Cancer Screening Program, Stage, Socioeconomic status, Disparity

## Abstract

**Background:**

The aim of this study was to evaluate whether stage at cancer diagnosis differed according to patient economic status.

**Methods:**

A total of 10,528 patients with cancer of the stomach, colorectum, breast, or cervix, which are target organs of the Korean National Cancer Screening Program (NCSP; fully implemented in 2005) were extracted from population-based cancer registries. The patients were classified into four groups based on socioeconomic status (SES), as determined using their National Health Insurance (NHI) monthly premium at the time of cancer diagnosis. Cancer stage at diagnosis was defined as early (in situ/local) or late stage (regional/distant) based on the Surveillance, Epidemiology, and End Results (SEER) summary stage. Multivariable logistic regression analysis was performed to estimate the risk of non-local stage using age, residential area, and community deprivation index as covariates.

**Results:**

The lowest SES subjects showed significantly higher risks of being diagnosed at a later stage for stomach, colorectal, and female breast cancer, but not for cervical cancer, compared with the highest SES subjects. The estimated ORs were 1.28 (95% CI, 1.10–1.49), 1.29 (95% CI, 1.03–1.61), and 1.35 (95% CI, 1.02–1.81) in the lowest SES subjects with stomach, colorectal, and breast cancer, respectively.

**Conclusions:**

In conclusion, later stage diagnoses of stomach, colon, and female breast cancer are still associated with SES in Korea in the era of the NCSP for the lower SES population.

## Highlights

• Advanced stage was more frequent in cancer patients with lower economic status.
• Differences of stage by SES were found in stomach, colorectal, and breast cancer.
• Disparities still exist after the National Cancer Screening program in Korea.

## Introduction

Disparity in cancer outcomes by socioeconomic status (SES) is a major global public health issue. Cancer stage at diagnosis is a critical determinant of cancer outcomes and is directly associated with survival in cancer patients. Several factors of SES, including race/ethnicity, insurance status, education level, and individual or neighborhood economic status, have been related to the risk of advanced stage cancer at diagnosis.^1^, ^2^, ^3^, ^4^, ^5^, ^6^ Individuals with low SES, individuals living in rural areas, and uninsured individuals are less likely to be regularly screened for cancer because of their limited access to medical care facilities. Thus, cancer patients with low SES, as assessed using income, education, or occupation, have a higher risk of advanced cancer at diagnosis^7^; however, evidence for the associations of SES with cancer stage at diagnosis according to primary site, ethnicity, and presence of national screening program is still inconsistent.^5^, ^6^, ^8^, ^9^, ^10^

In Korea, the National Cancer Screening Program (NCSP), which targets the population with lower economic status, was launched in 1999 and has subsequently been expanded to benefit more participants and screen more cancer sites. Since 2005, more than 15 million individuals who were Medical Aid (MA) recipients or National Health Insurance beneficiaries in the lower 50% of income level were provided free cancer screening for five major sites, including stomach, breast, cervix, colon, and liver. The NCSP was organized by the Korean National Health Insurance (NHI) Corporation, and the NCSP coverage rate among eligible populations in the target age range reached 50% in 2005.^11^ The Korean NHI is the country's only public insurance system and has been compulsory to all Korean residents and covered by all medical facilities since 1989. The NHI program is a co-payments and contributory program covering about 96% of the Korean population,^12^ and the MA program is a non-payable medical assistance program for people who are living under the national poverty line. The main financial sources of the programs are the premium from the insured of the NHI and government subsidy. The NHI premium is a useful proxy variable for the SES of Koreans because it is calculated based on employee's wages. In the case of self-employed Koreans, the premium is calculated based on the household income, assets, vehicles owned, and age of household members.^13^, ^14^

The objectives of this study were to determine the differences in cancer stage at diagnosis according to SES assessed using the NHI premium and to evaluate the disparities in cancer stage at diagnosis after full implementation of the NCSP in Korea.

## Methods

### Data sources

A total of 12,377 cases of stomach, colorectal, breast, and cervical cancer, which are target cancer sites for Korean NCSP, diagnosed during 2005–2007, were extracted from the Gwangju Cancer Registry (GCR) and Jeonnam Cancer Registry (JCR) databases. The GCR and JCR are population-based cancer registries that were established in 1997 and 2000, respectively, and have identical data collection and quality control processes.^15^ The Gwangju is a metropolitan city with 1.5 million population and Jeonnam is a representative rural province with 1.9 million population. The two registries cover approximately 6.7% of the Korean population. Of these cases, 794 cases (6.4%) were excluded because essential information, such as Surveillance, Epidemiology, and End Results (SEER) summary stage and NHIC premium amount each year, were not available. Seven male breast cancer cases were also excluded because of the different nature of male breast cancer from female breast cancer. Cancer cases diagnosed before the recommended screening age of each cancer site were also excluded (*n* = 1048). A total of 10,528 cases with stomach (5372 cases), colorectal (3128 cases), female breast (1323 cases), and cervical cancer (705 cases) were ultimately included in the analyses. All cases were classified as early stage (in-situ and local) or late stage (regional and distant stage) based on the SEER summary stage classification method.^16^ The proportion of histologically verified cases was 96.2%, and age was known for all cases. Proportion of Death Certificate Only (DCO) cases among the registered cases during 2005–2007 was 1.9%. DCO cases are not included the analysis. Individual information about attendance at screening programs and screen-detected cancer are not included in the standard dataset of the registries. This study was approved by the Institutional Review Board of Chonnam National University Hwasun Hospital (CNUHH-EXP-2015-068).

### Socioeconomic status

Individual-level SES was measured using average monthly insurance premiums imposed by the Korean NHIC at the year of cancer diagnosis, and all incident cancer cases were divided into quartiles based on the monthly premium. Individuals who were insured by the MA program were included in the lowest quartile group (*n* = 1106). Community-level SES was also measured using the deprivation index and rurality of residential areas. Deprivation indices of each administrative area, both material and social, were measured based on the 2005 Korean Census data, including nine household level and seven individual-level variables, as described in detail previously.^17^ Higher scores in each index indicate a greater level of deprivation. The scores were also categorized into quartiles. Residential areas were classified into four groups according to population number and adjacency to a metropolitan area, including metropolis (population of more than one million), urban (population of more than 100,000 and less than one million), rural (population of less than 100,000 and located near a metropolis), and remote rural areas (population of less than 100,000 and located distant from a metropolis).

### Statistical analyses

To evaluate the differences in later stage cancer risk according to SES, a multivariate logistic regression analysis was performed. The variables that were independently associated with the risk of later stage at diagnosis in the univariate logistic regression, age at cancer diagnosis, neighborhood deprivation index, and rurality of residential areas, were used as covariates for the adjusted model. Subjects in the highest SES group were used as the reference. The risk of later stage at diagnosis for each cancer site was calculated as an adjusted odds ratio (OR) with the corresponding 95% confidence interval (CI). Trend test in the adjusted model was used to identify the possible linear associations between the SES variables and risk of later stage at diagnosis. All analyses were performed using SAS statistical software (SAS Institute, Inc., Cary, NC, USA). All statistical tests were two-sided, and the level of statistical significance was 0.05.

## Results

Table 1 presents the distributions of the basic characteristics of the study subjects. Proportions of cases with stomach, colorectal, female breast, and cervix cancer diagnosed at late stage were 49.4%, 68.6%, 46.1%, and 40.2%, respectively. A total of 6462 cases (61.4%) were living in a metropolis or urban area, and 4066 cases (38.6%) were living in rural or remote rural areas at the time of cancer diagnosis (Table 1). The proportions of cases diagnosed at later stage according to rurality of residential area, community deprivation index, and amount of monthly premium of NHI are presented in eTable 1.

**Table 1:** Basic characteristics of study subjects according to cancer site (*n* = 10,528).

	Stomach (*n* = 5372)	Colorectal (*n* = 3128)	Breast (*n* = 1308)	Cervix (*n* = 705)
Sex				
Male	3629 (67.6)	1932 (61.8)	–	–
Female	1743 (32.4)	1196 (38.2)	1323 (100)	705 (100)
Quality index				
Histologic verification^a^	5099 (94.9)	2925 (93.5)	1298 (98.1)	699 (99.1)
Age at diagnosis, years				
<50	661 (12.3)	33 (1.1)	648 (49.0)	330 (46.8)
50-69	2805 (52.2)	1876 (60.0)	561 (42.4)	266 (37.7)
≥70	1906 (35.5)	1219 (39.0)	114 (8.6)	109 (15.5)
Calendar years at diagnosis				
2005	1910 (35.6)	1028 (32.9)	423 (32.0)	232 (32.9)
2006	1794 (33.4)	1019 (32.6)	439 (33.2)	264 (37.4)
2007	1668 (31.0)	1081 (34.6)	461 (34.8)	209 (29.6)
Rurality of residential area^b^				
Metropolitan area	1755 (32.7)	1211 (38.7)	624 (47.2)	287 (40.7)
Urban	1306 (24.3)	708 (22.6)	378 (28.6)	193 (27.4)
Rural	624 (11.6)	337 (10.8)	87 (6.6)	71 (10.1)
Remote rural	1687 (31.4)	872 (27.9)	234 (17.7)	154 (21.8)
SEER summary stage				
Early (In situ/Local)	2418 (45.0)	822 (26.3)	655 (49.5)	363 (51.5)
Late (Regional/Distant)	2954 (55.0)	2306 (73.7)	668 (50.5)	342 (48.5)
Amount of monthly premium				
Highest	1277 (23.8)	814 (26.0)	326 (24.6)	92 (13.0)
Mid-high	1378 (25.7)	796 (25.4)	352 (26.6)	165 (23.4)
Mid-low	1128 (21.0)	605 (19.3)	247 (18.7)	138 (19.6)
Lowest	1589 (29.6)	913 (29.2)	398 (30.1)	310 (44.0)
Community deprivation				
Lowest	1288 (24.0)	837 (26.8)	246 (18.6)	213 (30.2)
Mid-low	1431 (26.6)	630 (20.1)	449 (33.9)	169 (24.0)
Mid-high	1321 (24.6)	878 (28.1)	256 (19.4)	143 (20.3)
Highest	1332 (24.8)	783 (25.0)	372 (28.1)	180 (25.5)

SEER, Surveillance, Epidemiology, and End Results Program.

a Histological verification defined as cancer confirmation using cytology or pathologic diagnosis.

b Metropolis defined as city with a population of more than one million; urban defined as area with a population of more than 100,000 and less than one million; rural defined as area with a population of less than 100,000 and located near a metropolis; remote rural defined as area with populations of less than 100,000 and located distant from a metropolis.

The results from univariate logistic regression analyses are summarized in Table 2. The risks of late-stage cancer diagnosis in the eldest group (≥70 years) were significantly higher for stomach (OR 1.82; 95% CI, 1.52–2.18), female breast (OR 1.51; 95% CI, 1.01–2.26), and cervical cancer (OR 3.37; 95% CI, 2.11–5.40) compared with the youngest group (<50 years). No significant differences in the risk of later stage at diagnosis were observed for stomach or colorectal cancer according to sex. Subjects living in remote rural areas distant from metropolitan cities showed significantly increased risk of late-stage stomach cancer diagnosis (OR 1.18; 95% CI, 1.03–1.35). Subjects with the highest level of community deprivation showed a significant increased risk of late-stage stomach cancer (OR 1.18; 95% CI, 1.01–1.37) and colorectal cancer (OR 1.12; 95% CI, 1.07–1.23). Linear associations between the risk of late stage at cancer diagnosis and the quartiles of health insurance premium amount were found for stomach cancer and increased with lower premium amounts (P for trend = 0.003). The lowest-quartile health insurance premium group showed increased risks of later stage at diagnosis for stomach (OR 1.24; 95% CI, 1.07–1.43), colorectal (OR 1.23; 95% CI, 1.03–1.52), and female breast cancer (OR 1.35; 95% CI, 1.01–1.81) cases. No significant difference in risk of late stage at cancer diagnosis with insurance premium amount was found for cervical cancer cases (Table 2).

**Table 2:** Risk of advanced stage at the time of diagnosis by cancer site, univariable logistic regression.

	Odds ratios (95% confidence intervals)			
	Stomach (*n* = 5372)	Colorectal (*n* = 3128)	Breast (*n* = 1308)	Cervix (*n* = 705)
Age, years				
<50	1 (reference)	1 (reference)	1 (reference)	1 (reference)
50-69	0.93 (0.78–1.10)	1.43 (0.70–2.92)	1.01 (0.80–1.26)	1.15 (0.83–1.60)
≥70	1.82 (1.52–2.18)	1.98 (0.96–4.08)	1.51 (1.01–2.26)	3.37 (2.11–5.40)
Sex				
Female	1 (reference)	1 (reference)		
Male	1.07 (0.95–1.20)	0.99 (0.84–1.17)		
Rurality^a^				
Metropolis	1 (reference)	1 (reference)	1 (reference)	1 (reference)
Urban	1.03 (0.89–1.19)	1.01 (0.81–1.24)	1.02 (0.87–1.45)	1.08 (0.75–1.25)
Rural	1.08 (0.90–1.30)	0.95 (0.72–1.25)	1.31 (0.84–2.06)	0.74 (0.44–1.26)
Remote rural	1.18 (1.03–1.35)	0.92 (0.76–1.13)	1.20 (0.89–1.62)	1.44 (0.97–2.14)
Community deprivation index				
Lowest	1 (reference)	1 (reference)	1 (reference)	1 (reference)
Mid-low	0.99 (0.85–1.15)	1.03 (0.81–1.30)	0.77 (0.56–1.06)	1.12 (0.75–1.68)
Mid-high	1.17 (1.00–1.36)	0.87 (0.71–1.08)	0.88 (0.61–1.25)	1.20 (0.79–1.84)
Highest	1.18 (1.01–1.37)	1.12 (1.07–1.23)	0.94 (0.67–1.30)	1.33 (0.90–1.98)
Amount of monthly premium				
Highest	1 (reference)	1 (reference)	1 (reference)	1 (reference)
Mid-high	1.06 (0.91–1.23)	1.05 (0.84–1.30)	1.07 (0.79–1.44)	1.02 (0.61–1.70)
Mid-low	1.06 (0.90–1.25)	0.94 (0.75–1.19)	0.99 (0.72–1.39)	1.08 (0.63–1.82)
Lowest	1.24 (1.07–1.43)	1.23 (1.03–1.52)	1.35 (1.01–1.81)	1.13 (0.71–1.79)

a Metropolis defined as city with a population of more than one million; urban defined as area with a population of more than 100,000 and less than one million; rural defined as area with a population of less than 100,000 and located near a metropolis; remote rural defined as area with populations of less than 100,000 and located distant from a metropolis.

Table 3 presents the results of the multivariate logistic regression analysis, with age group, rurality, community deprivation, and individual monthly premium as covariates. Linear associations between the risk of late-stage cancer diagnosis and insurance premium quartiles were found for stomach and colorectal cancer (P for trend = 0.001 and 0.041, respectively). For female breast cancer, borderline significance was shown for the linear association. The lowest premium class showed a higher risk of late-stage stomach (OR 1.28; 95% CI, 1.10–1.49), colorectal (OR 1.29; 95% CI, 1.03–1.61), and female breast cancer (OR 1.35; 95% CI, 1.02–1.81) (Table 3).

**Table 3:** Association between socioeconomic status and risk of advanced stage at time of diagnosis by cancer site, multivariable logistic regression.^a^

	Odds ratios (95% confidence intervals)			
	Stomach (*n* = 5372)	Colorectal (*n* = 3128)	Breast (*n* = 1308)	Cervix (*n* = 705)
Rurality^b^				
Metropolis	1 (reference)	1 (reference)	1 (reference)	1 (reference)
Urban	1.08 (0.86–1.36)	1.06 (0.79–1.41)	1.49 (0.98–2.26)	0.90 (0.52–1.56)
Rural	0.85 (0.64–1.14)	0.85 (0.56–1.28)	1.26 (0.65–2.24)	0.41 (0.17–1.03)
Remote rural	0.94 (0.70–1.26)	0.81 (0.56–1.18)	1.08 (0.61–1.91)	1.14 (0.70–1.83)
*P for trend*	0.854	0.230	0.366	0.992
Community deprivation index				
Lowest	1 (reference)	1 (reference)	1 (reference)	1 (reference)
Mid-low	0.96 (0.83–1.12)	1.05 (0.76–1.41)	0.77 (0.56–1.06)	1.09 (0.73–1.65)
Mid-high	1.16 (0.94–1.43)	0.91 (0.71–1.16)	0.89 (0.62–1.27)	1.27 (0.80–2.01)
Highest	1.18 (0.90–1.56)	1.05 (0.78–1.42)	0.95 (0.59–1.52)	1.52 (0.81–2.82)
*P for trend*	0.657	0.710	0.198	0.193
Amount of monthly premium				
Highest	1 (reference)	1 (reference)	1 (reference)	1 (reference)
Mid-high	1.12 (0.96–1.30)	1.11 (0.89–1.39)	1.04 (0.76–1.41)	1.08 (0.64–1.82)
Mid-low	1.15 (0.97–1.35)	1.05 (0.83–1.33)	1.01 (0.72–1.41)	1.18 (0.69–2.02)
Lowest	1.28 (1.10–1.49)	1.29 (1.03–1.61)	1.35 (1.02–1.81)	1.16 (0.72–1.87)
*P for trend*	0.001	0.041	0.051	0.531

a Age, sex, rurality of residential area, and community deprivation index were included as covariates.

b Metropolis defined as city with a population of more than one million; urban defined as area with a population of more than 100,000 and less than one million; rural defined as area with a population of less than 100,000 and located near a metropolis; remote rural defined as area with populations of less than 100,000 and located distant from a metropolis.

## Discussion

In this study, the impact of individual- and community-level SES on the late-stage cancer diagnosis of stomach, colorectal, female breast, and cervical cancer was evaluated using population-based cancer registry data. No overall difference in the risk of later stage at diagnosis according to community-level SES, as indicated by deprivation index and rurality of neighborhood, was found. However, significantly increased risks of later stage at diagnosis were found for stomach, colorectal, and female breast cancer according to individual-level SES, as indicated by the Korean NHIC monthly premium.

The Korean NCSP, a free cancer screening program organized by the Korean NHIC and Korean Ministry of Health and Welfare, was launched in 1999. Since 2004, the NCSP has screened free-of-charge for five major cancers (stomach, colorectal, female breast, cervix, and liver cancer), and it targets the population with relatively lower income levels, which are assessed using the Korean NHIC premium. The NCSP for stomach cancer, the most common cancer in Korean population, is aimed at those aged 40 years or over, who are invited to medical facilities to receive a gastro-endoscopy or upper gastrointestinal series every 2 years. Annual fecal occult blood test and biannual mammography and Pap smear are recommended to those aged ≥50 years, ≥40 years, and ≥30 years, respectively. To screen for liver cancer, ultrasound scanning and alpha-fetoprotein test are done for those aged ≥40 years with hepatitis B or hepatitis C. All target subjects receive invitation letters from the Korean NHIC and health departments of their administrative district. Currently, the proportion of beneficiaries of Korean NCSP has expanded to half of the target population in the recommended screening age ranges since 2005. During the study period, summary coverage rates of NCSP among the total Korean population of eligible age were 55.8%, 51.2%, 57.5%, and 53.0% for stomach, colorectal, breast, and cervix cancer, respectively.^11^ Despite the recent quantitative expansion of the NCSP, no evidence for the effect of the nationwide program has been established. Some challenging issues for the NCSP, such as the low participation rate among the eligible population, low positive predictive rate, and low sensitivity of screening checkups, are still present.^11^, ^18^, ^19^ Moreover, income inequalities in screening attendance still exist because of various barriers that prevent the low-income population from attending cancer screening.^18^, ^20^ The respective participation rates in the NCSP for stomach, colorectal, breast, and cervix cancer were 22.5%, 16.5%, 27.1%, and 23.9% during 2005–2007, and these attendance rates of NCSP subjects were lower than those of the total Korean population.^11^ Therefore, the present study was designed to evaluate the differences in the risk of later stage at diagnosis according to SES in the NCSP era of South Korea. The impact of the NCSP was indirectly evaluated by examining whether stage at diagnosis was different among the lower SES populations, the main target population of the NCSP. We found an overall difference in the proportion of late stage at diagnosis according to individual SES and a significantly higher risk of later stage at diagnosis in the lowest SES for all cancers being screened for, except cervical cancer. These results suggest that socio-economic disparities still affect cancer diagnosis, despite the extensive national support for low SES populations, including organized cancer screening programs and financial support.

Multiple previous studies have shown SES-related disparities in cancer diagnosis. For stomach cancer, studies have shown that SES is an independent prognostic factor for survival.^21^, ^22^ However, to date, only one study has examined the association between SES and stage at diagnosis for stomach cancer, and no association was found.^23^ In our study, a strong inverse association between SES and stage at diagnosis for stomach cancer was found (P for trend = 0.001), and patients in the lowest SES had the highest risk of later stage at diagnosis (OR 1.28; 95% CI, 1.10–1.49). This association persisted in sensitivity analyses, after re-categorizing the MA group as an independent group of lowest SES (eTable 2). Many epidemiologic studies have also investigated SES-related disparities in stage at diagnosis of colon, female breast, and cervical cancer, but findings for different sites and races/ethnicities have been inconsistent.^1^, ^2^, ^4^, ^5^, ^8^, ^23^ Considering the inconsistent findings and potential residual confounders in the previous studies, more factors still need to be evaluated to clarify the association between SES and stage at cancer diagnosis for various sites and under different social environments. In the present study, female breast and colorectal cancer patients in the lowest SES had significantly higher risks of later stage at diagnosis than patients in the highest SES (OR 1.35; 95% CI, 1.02–1.81 and OR 1.29; 95% CI, 1.03–1.61, respectively). For cervical cancer, women in the lower-SES group had a higher risk of later stage at diagnosis because they were less likely to receive Pap tests^18^, ^24^ and had longer delays between the development of symptoms and cancer diagnosis.^25^ However, disparities related to SES among patients with cervical cancer were not found in this study, perhaps because the proportion of cervical cancer patients with earlier stage at diagnosis was relatively higher than proportions for other cancer types, which may be related to the high accessibility of the Pap smear test and the oncological units used for cervical cancer, regardless of a patient's SES. In addition, cervical cancer screening can detect precancerous lesions and some preinvasive carcinomas occurring in the uterine cervix. These characteristics of cervix cancer screening may attenuate the potential disparities of cancer stage at diagnosis according to SES.

SES can be measured by various tools at the individual, household, and community levels. In this study, monthly premium amount imposed by the Korean NHIC was used to assess individual SES. The NHIC premium is a useful proxy variable for SES because it is considered a general property tax in Korea and is calculated based on an individual's income level, occupation, and household assets.^13^, ^14^ The Korean population was classified based on economic status and jobs into three categories: medical aid, self-employed insured, and employee-insured. The premium levels are calculated differently for the self-employed and employee categories, but stratification by these categories was not considered in the data analyses.

Living in rural areas also has been suggested as a potential risk factor for later-stage cancer diagnosis, possibly because of the limited number of cancer screening facilities and poor accessibility to those facilities in rural areas.^6^, ^26^ In our study, no significant association between rurality and risk of later stage at diagnosis was found. This may be due to the geographic characteristics of Korean rural areas. In Korea, most rural areas, even remote rural areas, are located within 2 h of a metropolis, and cancer screening mobile units, which are equipped for mammography and upper gastro-intestinal series, are commonly available, even in remote rural areas. Mobile units provide underserved residents the opportunity to receive cancer screening services when needed. Thus, the participation rate in the cancer screening program between urban and rural areas may not have been different.

The present study had some limitations. First, our analyses did not consider changes in stage distributions before and after the NCSP was launched because no data were available on stage distributions before introduction of the NCSP. Our results suggest a null effect of NCSP on cancer stage shift, but this finding is not yet conclusive. Second, the three categories of the NSCP eligible populations (MA, employee, and self-employed) were not stratified,^13^, ^14^ and this may have caused some misclassification of SES. This limitation may have affected our results, but the effect is likely to be non-differential and toward the null direction. Third, the data were derived from two cancer registries covering approximately 6.7% of the total Korean population. Since the data may not be representative of the whole Korean population, we selected the registries that cover both metropolitan and rural area to minimize selection bias. Fourth, more individualized information about the screening attendance or status of screen-detected cancer could not be used in the analysis. Fifth, NCSP participation rates among the target population during the study period, 2005–2007, were quite low (below 30%)^11^; thus, more long-term evaluation of more cases is needed to clearly determine whether the risk of advanced stage is different according to SES. Last, population by SES group as the denominator of stage-specific incidence was not available in this study, so stage frequencies among the patients were used instead. Difference of stage proportion between the SES groups is not enough to provide conclusive results. In conclusion, similar to the results of some previous studies, inverse relationships between SES and risks of later stage at diagnosis for stomach, colorectal, and female breast cancer were found, and these results suggest that socioeconomic disparities are still present in cancer diagnosis, despite the nationwide screening program. Increasing the NCSP participation rate and improving the quality of the program may be necessary to accomplish the mission of the NCSP, which is mainly to reduce disparities in cancer diagnosis.

## Conflicts of interest

None declared.

## References

[1] Hahn K.M., Bondy M.L., Selvan M. (2007). Factors associated with advanced disease stage at diagnosis in a population-based study of patients with newly diagnosed breast cancer. Am J Epidemiol.

[2] Halpern M.T., Pavluck A.L., Ko C.Y., Ward E.M. (2009). Factors associated with colon cancer stage at diagnosis. Dig Dis Sci.

[3] Wells B.L., Horm J.W. (1992). Stage at diagnosis in breast cancer: race and socioeconomic factors. Am J Public Health.

[4] Clegg L.X., Reichman M.E., Miller B.A. (2009). Impact of socioeconomic status on cancer incidence and stage at diagnosis: selected findings from the surveillance, epidemiology, and end results: national Longitudinal Mortality Study. Cancer Causes Control.

[5] Flores Y.N., Davidson P.L., Nakazono T.T., Carreon D.C., Mojica C.M., Bastani R. (2013). Neighborhood socio-economic disadvantage and race/ethnicity as predictors of breast cancer stage at diagnosis. BMC Public Health.

[6] Nguyen-Pham S., Leung J., McLaughlin D. (2014). Disparities in breast cancer stage at diagnosis in urban and rural adult women: a systematic review and meta-analysis. Ann Epidemiol.

[7] Byers T.E., Wolf H.J., Bauer K.R. (2008). The impact of socioeconomic status on survival after cancer in the United States : findings from the National Program of Cancer Registries Patterns of Care Study. Cancer.

[8] El Ibrahimi S., Pinheiro P. (2015). No differences in cervical cancer stage at diagnosis for Blacks and Whites in the mountain west. J Immigr Minor Health.

[9] Chatterjee N.A., He Y., Keating N.L. (2013). Racial differences in breast cancer stage at diagnosis in the mammography era. Am J Public Health.

[10] Sassi F., Luft H.S., Guadagnoli E. (2006). Reducing racial/ethnic disparities in female breast cancer: screening rates and stage at diagnosis. Am J Public Health.

[11] Kim Y., Jun J.K., Choi K.S., Lee H.Y., Park E.C. (2011). Overview of the National Cancer Screening Programme and the cancer screening status in Korea. Asian Pac J Cancer Prev.

[12] Song Y.J. (2009). The South Korean health care system. Jpn Med Assoc J.

[13] Joshi S., Song Y.M., Kim T.H., Cho S.I. (2008). Socio-economic status and the risk of liver cancer mortality: a prospective study in Korean men. Public Health.

[14] Song Y.M., Ferrer R.L., Cho S.I., Sung J., Ebrahim S., Davey Smith G. (2006). Socioeconomic status and cardiovascular disease among men: the Korean national health service prospective cohort study. Am J Public Health.

[15] Kweon S.S., Shin M.H., Chung I.J., Kim Y.J., Choi J.S. (2013). Thyroid cancer is the most common cancer in women, based on the data from population-based cancer registries, South Korea. Jpn J Clin Oncol.

[16] Young J.L., Bethesda M.D. (2006). SEER Summary Staging Manual, 2000: Codes and Coding Instructions.

[17] Choi M.H., Cheong K.S., Cho B.M. (2011). Deprivation and mortality at the town level in Busan, Korea: an ecological study. J Prev Med Public Health.

[18] Park M.J., Park E.C., Choi K.S., Jun J.K., Lee H.Y. (2011). Sociodemographic gradients in breast and cervical cancer screening in Korea: the Korean National Cancer Screening Survey (KNCSS) 2005–2009. BMC Cancer.

[19] Jung M. (2015). National Cancer Screening Programs and evidence-based healthcare policy in South Korea. Health Policy.

[20] Kim S., Kwon S., Subramanian S.V. (2015). Has the National Cancer Screening Program reduced income inequalities in screening attendance in South Korea?. Cancer Causes Control.

[21] Siemerink E.J., Hospers G.A., Mulder N.H., Siesling S., van der Aa M.A. (2011). Disparities in survival of stomach cancer among different socioeconomic groups in North-East Netherlands. Cancer Epidemiol.

[22] Yu X.Q., O'Connell D.L., Gibberd R.W., Armstrong B.K. (2008). Assessing the impact of socio-economic status on cancer survival in New South Wales, Australia 1996–2001. Cancer Causes Control.

[23] Gornick M.E., Eggers P.W., Riley G.F. (2004). Associations of race, education, and patterns of preventive service use with stage of cancer at time of diagnosis. Health Serv Res.

[24] Coughlin S.S., King J., Richards T.B., Ekwueme D.U. (2006). Cervical cancer screening among women in metropolitan areas of the United States by individual-level and area-based measures of socioeconomic status, 2000 to 2002. Cancer Epidemiol Biomarkers Prev.

[25] Berraho M., Obtel M., Bendahhou K. (2012). Sociodemographic factors and delay in the diagnosis of cervical cancer in Morocco. Pan Afr Med J.

[26] Parikh-Patel A., Bates J.H., Campleman S. (2006). Colorectal cancer stage at diagnosis by socioeconomic and urban/rural status in California, 1988–2000. Cancer.

