# Supplementary material for: Difference of stage at cancer diagnosis by socioeconomic status for four target cancers of the National Cancer Screening Program in Korea: Results from the Gwangju and Jeonnam cancer registries
Source: J Epidemiol. 2017 Mar 6;27(7):299–304. doi: 10.1016/j.je.2016.07.004 (PMC5498418; doi:10.1016/j.je.2016.07.004)
Supplement: Supplementary file 1 [file mmc1.pdf]

eTable 1. Proportion of later stage (regional/distant) at diagnosis according to socioeconomic status

	Stomach (n=5,372)	Colorectal (n=3,128)	Breast (n=1,308)	Cervix (n=705)
Rurality of residential area				
Metropolitan area	935 (53.3)	899 (74.2)	302 (48.4)	134 (46.7)
Urban	706 (54.1)	526 (74.3)	194 (51.3)	94 (48.7)
Rural	345 (55.3)	247 (73.3)	48 (55.2)	28 (39.4)
Remote rural	968 (57.4)	634 (72.7)	124 (53.0)	86 (55.8)
Community Deprivation				
Lowest	684 (53.1)	623 (74.4)	123 (53.2)	96 (45.1)
Mid-low	756 (52.8)	472 (74.9)	210 (46.8)	81 (47.9)
Mid-high	752 (56.9)	630 (71.8)	128 (50.0)	71 (49.7)
Highest	761 (57.1)	581 (74.2)	192 (51.6)	94 (52.2)
Amount of monthly premium				
Highest	674 (52.8)	591 (72.6)	156 (47.9)	43 (46.7)
Mid-high	746 (54.1)	585 (73.5)	174 (49.4)	78 (47.3)
Mid-low	612 (54.3)	432 (71.4)	118 (47.8)	67 (48.6)
Lowest	922(58.0)	698 (76.5)	220 (55.3)	154 (49.7)

eTable 2. Sensitivity analysis of the association between the risk of later stage at diagnosis and amount of monthly premium

	Odds ratios (95% confidence intervals)			
	Stomach (n=5,372)	Colorectal (n=3,128)	Breast (n=1,308)	Cervix (n=705)
Amount of monthly [illegible]				
Highest	1 (reference)	1 (reference)	1 (reference)	1 (reference)
Mid-high	1.12 (0.96-1.30)	1.11 (0.89-1.39)	1.04 (0.76-1.41)	1.08 (0.64-1.82)
Mid-low	1.15 (0.97-1.35)	1.05 (0.83-1.33)	1.01 (0.72-1.41)	1.18 (0.69-2.02)
Lowest	1.28 (1.08-1.52)	1.40 (1.09-1.80)	1.13 (0.82-1.57)	1.06 (0.64-1.76)
Medical Aid	1.28 (1.05-1.57)	1.09 (0.81-1.48)	2.00 (1.30-3.07)	1.39 (0.78-2.36)
P for trend	0.002	0.076	0.013	0.399
